# 613 cases of splenic rupture without risk factors or previously diagnosed disease: a systematic review

**DOI:** 10.1186/1471-227X-12-11

**Published:** 2012-08-14

**Authors:** F Kris Aubrey-Bassler, Nicholas Sowers

**Affiliations:** 1 Primary Healthcare Research Unit, Memorial University of Newfoundland, Health Sciences Centre, St. John’s, Newfoundland and Labrador, St Johns, Canada; 2 Discipline of Emergency Medicine, Memorial University of Newfoundland, St. John’s, Newfoundland and Labrador, St Johns, Canada; 3 Discipline of Family Medicine, Memorial University of Newfoundland, St. John’s, Newfoundland and Labrador, St Johns, Canada; 4 Department of Emergency Medicine, Dalhousie University, Halifax, NS, Canada

**Keywords:** Splenic rupture, Rupture, Spontaneous, Atraumatic, Presenting complaint, Initial manifestation

## Abstract

**Background:**

Rupture of the spleen in the absence of trauma or previously diagnosed disease is largely ignored in the emergency literature and is often not documented as such in journals from other fields. We have conducted a systematic review of the literature to highlight the surprisingly frequent occurrence of this phenomenon and to document the diversity of diseases that can present in this fashion.

**Methods:**

Systematic review of English and French language publications catalogued in Pubmed, Embase and CINAHL between 1950 and 2011.

**Results:**

We found 613 cases of splenic rupture meeting the criteria above, 327 of which occurred as the presenting complaint of an underlying disease and 112 of which occurred following a medical procedure. Rupture appeared to occur spontaneously in histologically normal (but not necessarily normal size) spleens in 35 cases and after minor trauma in 23 cases. Medications were implicated in 47 cases, a splenic or adjacent anatomical abnormality in 31 cases and pregnancy or its complications in 38 cases.

The most common associated diseases were infectious (n = 143), haematologic (n = 84) and non-haematologic neoplasms (n = 48). Amyloidosis (n = 24), internal trauma such as cough or vomiting (n = 17) and rheumatologic diseases (n = 10) are less frequently reported. Colonoscopy (n = 87) was the procedure reported most frequently as a cause of rupture. The anatomic abnormalities associated with rupture include splenic cysts (n = 6), infarction (n = 6) and hamartomata (n = 5). Medications associated with rupture include anticoagulants (n = 21), thrombolytics (n = 13) and recombinant G-CSF (n = 10). Other causes or associations reported very infrequently include other endoscopy, pulmonary, cardiac or abdominal surgery, hysterectomy, peliosis, empyema, remote pancreato-renal transplant, thrombosed splenic vein, hemangiomata, pancreatic pseudocysts, splenic artery aneurysm, cholesterol embolism, splenic granuloma, congenital diaphragmatic hernia, rib exostosis, pancreatitis, Gaucher's disease, Wilson's disease, pheochromocytoma, afibrinogenemia and ruptured ectopic pregnancy.

**Conclusions:**

Emergency physicians should be attuned to the fact that rupture of the spleen can occur in the absence of major trauma or previously diagnosed splenic disease. The occurrence of such a rupture is likely to be the manifesting complaint of an underlying disease. Furthermore, colonoscopy should be more widely documented as a cause of splenic rupture.

## Background

Rupture of the spleen is relatively common both immediately and in a delayed fashion following significant blunt abdominal injury [1], and this phenomenon is well documented in the scientific literature and textbooks (e.g. [2,3]). While less common, cases of atraumatic rupture of diseased spleens are also widely reported in the literature (reviewed in [4,5]). In contrast, the phenomenon of splenic rupture in the absence of these two risk factors is not documented in emergency medicine textbooks [2,3] and we believe that it is not widely appreciated by emergency physicians.

Cases of splenic rupture not fitting the description above are related by their lack of historical cues to suggest the diagnosis at presentation. This distinguishes them from other causes of splenic rupture and highlights the importance to emergency physicians who rely a great deal on the patient history to appropriately triage patients for definitive investigation and referral. A recent systematic review of cases of atraumatic rupture of the spleen has been published [4]; however, a surprising number of the splenic rupture cases reported in this review and elsewhere represent the presenting complaint of the underlying disease process. The authors of the review do not highlight this fact which we believe to be crucial information to the practicing clinician. Therefore, we have reviewed the literature on cases of splenic rupture for which there was *not* an immediately obvious cause apparent on presentation such as significant trauma (either recent or remote) or previously diagnosed disease known to affect the spleen.

## Methods

We conducted a systematic review of English and French language papers indexed in CINAHL, PubMed and Embase using the medical subject heading (MeSH) search terms “rupture, spontaneous,” and “splenic rupture,” (or equivalent for the different databases) combined with the textword search “undiagnosed” or “first manifestation” or “presenting” or “spontaneous.” This search strategy was combined with an additional strategy including the MeSH terms “rupture, spontaneous” and “spleen” and the free text “normal spleen;” both strategies were used together to extract relevant papers. Searches were limited to English and French language papers on human subjects published in the years 1950 to 2011. We also explored multiple other textword modifiers such as “atraumatic,” “non-traumatic” and “trivial,” none of which improved the sensitivity of the search with sufficient specificity to be helpful. Searches were developed by a research librarian and one of the authors who has training in clinical epidemiology (KA).

The reference lists of the papers so identified were also examined for relevant additions. We elected to include papers written in other languages if an English language abstract was available that included the information necessary for our report. Because the information we were trying to extract was fairly straightforward, we elected to include cases from papers for which only the abstract was available to us if the necessary information was reported there.

Case reports and case series’ were examined for relevance. Data was extracted from cases referenced in review papers only if the original paper was not available to us, and these were cross-referenced with case reports to prevent duplicate recording. Papers pertaining to the rupture of diseased spleens were excluded if the disease was correctly diagnosed prior to presentation at the emergency department. Cases of splenic rupture occurring immediately following any trauma (including trivial) were also excluded. Delayed splenic rupture cases were excluded if they occurred greater than 48 hours after major trauma (because this phenomenon is well reported in the literature and textbooks), but were included if the inciting traumatic event was considered by the two authors to be of trivial severity. Although the degree of trauma is debatable, we elected to include cases likely caused by cough or vomiting because we felt that these aetiologic factors were also under-appreciated. Although delayed post-medical procedure rupture of the spleen is documented in the proceduralist (surgical and GI) literature, it is not documented in EM textbooks and we have elected to include these cases here. We limited our report to papers published since 1950. Although the diagnosis and treatment of splenic rupture has changed considerably in recent years, we found no evidence to suggest that the underlying causes of rupture have changed during this time period. Because the primary purpose of our paper was to highlight aetiology and not diagnosis or management, we elected to choose a somewhat broader time period than might have been appropriate for a study with a different purpose. The information extracted onto a spreadsheet included the splenic disease process if any, other evidence of splenic abnormality (anatomical or histological), and the nature of any associated trauma. Causative processes were grouped into clinically relevant categories. We did not attempt to document histological or pathological findings, or review diagnostic or treatment methods as these are recently reviewed in detail elsewhere [4,5].

## Results

**Figure 1:**
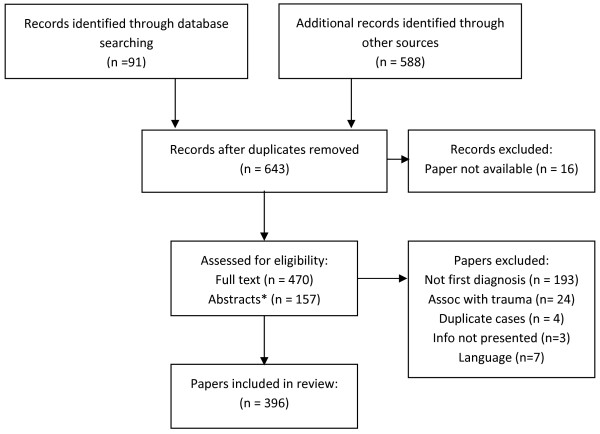
**PRISMA **[7]** flow diagram documenting number of references processed.** Legend: *Only abstracts containing all necessary information were included. Abbreviations: Assoc = associated.

**Figure 2:**
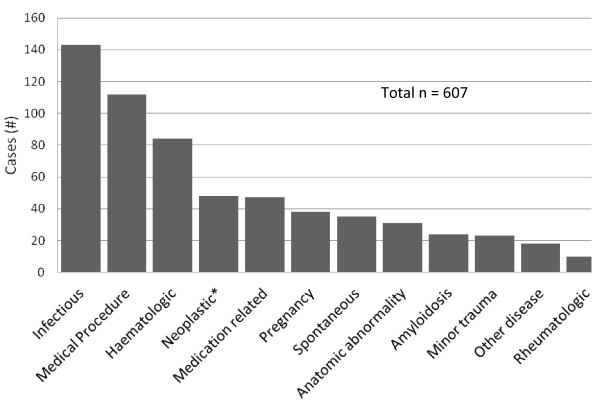
**Categorization of all splenic rupture cases meeting inclusion criteria.** Legend: * Non-haematologic only; haematologic neoplasms are contained in the haematologic category.

No Medical Subject Headings or other keywords reliably identified the 396 papers reporting 607 cases of splenic rupture that met our inclusion criteria. Thus, we manually reviewed many abstracts and papers that ended up being excluded from this review (Figure 1). Some case series referenced here report both cases meeting our inclusion criteria and others meeting our exclusion criteria; only those meeting the inclusion criteria are included. We attempted to obtain all of the original papers referenced here so that we could document the cases without relying on secondary sources. However, sixteen of the papers were not accessible to us nor were we able to find the information necessary to fully ascertain whether the cases described within them were appropriate for this review. All cases are categorized in Figure 2 and clinically relevant sub-categories are presented in Tables 1, 2, 3, 4, 5, 6.

**Table 1:** Cases of splenic rupture following a medical procedure

**Procedure**	**<48 hours**	**References**	**>48 hours**	**References**
Colonoscopy	79	[8-20]	8	[10,11,21-23]
ERCP	4	[24-27]	4	[28-31]
Liver surgery	5	[32-36]		
Pulmonary Surgery	2	[37,38]		
Gastroscopy			1	[39]
Displaced CAPD catheter			1	[40]
ESWL	1	[41]		
ECT	1	[42]		
Abdominal surgery	1	[43]		
Laparoscopy	1	[44]		
Hysterectomy	4	[45-48]		
**TOTAL**	**98**		**14**	

**Table 2:** Cases of splenic rupture associated with previously undiagnosed infiltrative or inflammatory pathology

**Disease**		**Cases**	**References**
**Infectious**			
	Malaria	65	[49-60]
	Mononucleosis	42	[61-90]
	CMV	6	[91-95]
	Typhoid fever	4	[96,97]
	Endocarditis with splenic embolism	3	[98-100]
	Pneumonia	3	[101-103]
	HIV	3	[104-106]
	Q fever	2	[107,108]
	Salmonella sp.	2	[109,110]
	Splenic tuberculosis	2	[111,112]
	Viral Hepatitis	2	[113,114]
	EBV	1	[115]
	Babesiosis	1	[116]
	Brucellosis	1	[117]
	Bartonella	1	[118]
	Dengue fever	1	[119]
	Enterobacter cloacae	1	[120]
	Murine typhus	1	[121]
	Rickettsia sp.	1	[122]
	Varicella	1	[123]
**Haematologic**			
	Non-Hodgkin’s Lymphoma	25	[124-145]
	Hodgkin’s Lymphoma	5	[146-150]
	Undifferentiated Lymphoma	3	[151-153]
	Acute Lymphoblastic Leukemia	18	[154-165]
	Chronic Myelogenous Leukemia	7	[166-172]
	Acute Myelogenous Leukemia	6	[64,173-177]
	Hairy Cell Leukemia	5	[178-182]
	Acute T-cell Leukemia	1	[183]
	Undfifferentiated Leukemia	1	[184]
	Histiocytosis	6	[185-190]
	Multiple Myeloma	2	[63,191]
	Idiopathic Thrombocytic Purpura	1	[192]
	Myelofibrosis	1	[193]
	Polycythemia Vera	1	[194]
	Sickle Cell Disease	1	[195]
	Essential Thrombocythemia	1	[196]
**Rheumatologic**			
	Wegener’s Granulomatosis	3	[197-199]
	Polyarteritis Nodosa	3	[200,201]
	Systemic Lupus Erythematosis	3	[202-204]
	Rheumatoid Arthritis	1	[205]
**Other**			
	Amyloidosis	24	[65,206-216]
	Peliosis	8	[217-224]
	Pancreatitis	5	[151,225-228]
	Gaucher’s Disease	1	[80]
	Wilson’s Disease	1	[229]
**Total**		**276**	

**Table 3:** Cases of splenic rupture associated with the first diagnosis of a splenic or adjacent physical abnormality

**Diseases**	**Cases**	**References**
Splenic cyst	6	[6,61,230-233]
Splenic infarction	6	[234-238]
Splenic hamartoma	5	[239-243]
Hemangioma	3	[244-246]
Pancreatic pseudocyst	2	[247,248]
Thrombosed splenic vein	2	[249,250]
Splenic artery aneurysm	1	[251]
Empyema	1	[252]
Remote Pancreato-Renal Transplant	1	[253]
Cholesterol Embolism	1	[254]
Splenic granuloma	1	[255]
Congenital Diaphragmatic Hernia	1	[256]
Rib exostosis	1	[257]
**TOTAL**	**31**	

**Table 4:** Pregnancy related causes of splenic rupture

**Cause**	**Cases**	**References**
Normal pregnancy	22	[257-278]
Splenic ectopic pregnancy	9	[279-287]
Post-vaginal delivery	2	[288,289]
Post-caesarean section	2	[290,291]
Preeclampsia	1	[292]
Ruptured ectopic pregnancy	1	[225]
Non-splenic		
HELLP syndrome	1	[293]
**Total**	**38**	

Abbreviations: *HELLP*: Hemolysis, Elevated Liver Enzymes, Low Platelets.

**Table 5:** Previously undiagnosed, non-hematologic neoplastic causes of spontaneous splenic rupture

**Cause**	**Cases**	**References**
Angiosarcoma	31	[294-309]
Choriocarcinoma	5	[310-315]
Pancreatic cancer	4	[316-319]
Gastric cancer	1	[320]
Lung cancer	4	[313,321-323]
Kaposi Sarcoma	1	[324]
Granulosa cell tumour	1	[325]
Myofibroblastic tumour	1	[326]
**Total**	**48**	

**Table 6:** Other cases of splenic rupture

**Cause**		**Cases**	**References**
**Medication related**			
	Anticoagulant	21	[327-337]
	Thrombolytic	13	[83,329,338-342]
	Recombinant G-CSF	10	[343-352]
	Anti-platelet agents	2	[353]
	HIT	1	[354]
**Internal trauma-related**			
	Cough	12	[98,151,355-364]
	Vomiting	4	[365-368]
	Seizure	1	[249]
**Miscellaneous**			
	Spontaneous	35	[43,61,63-65,80,113,225,249,369-391]
	>48h after minor external trauma	6	[249,392-396]
	Pheochromocytoma	2	[397,398]
	Afibrinogenemia	1	[399]
**TOTAL**		**108**	

Abbreviations: G-CSF: Granulocyte Colony Stimulating Factor; HIT: Heparin Induced Thrombocytopenia.

## Discussion

Although rupture of the spleen in the absence of previously diagnosed disease or trauma is widely described as rare, given the extensive reports in the literature documented here, we believe that this descriptor should no longer be used. Although its existence is debated [1,369,400-402], sufficient reports from multiple authors are available to strongly suggest that rupture can occur spontaneously in otherwise normal spleens, but that this phenomenon is very rare. Given these two facts, the emergency clinician must be attuned to the possibility of splenic rupture in patients presenting with compatible symptoms without a compatible history. ED physicians must also be aware that such a presentation is very likely to be the manifesting episode of an underlying disease or anatomical abnormality. In the only other reference to these surprising findings, Renzulli found that the underlying cause for 51.2 % of the cases of atraumatic splenic rupture was not elicited until after hospital presentation [4].

In 1958, Orloff and Peskin proposed four criteria to define a true spontaneous rupture of a spleen [206], which emphasize that the spleen must appear grossly and histologically normal. In the same paper, they cite 71 reports documenting ruptures of the spleen labelled as spontaneous, only 20 of which fulfilled all of their criteria. Thus, usage of the term spontaneous was inconsistent and continues to be so in the more recent literature, with many authors labeling the rupture of diseased spleens as spontaneous. We highlight this because many of the pathological ruptures that we have documented here (as well as pathological ruptures in patients with previously known disease documented elsewhere [6]) include the word spontaneous in the title and no information on the associated pathology [8,61,91,98,124,151,154,355-357,365,400,403]. Thus, readers skimming titles may be mistaken in thinking that true spontaneous rupture is more common than thought.

As we have shown here, documentation of rupture of the spleen following colonoscopy is relatively common with at least 87 cases reported (Table 2). However, we found only 1 such case reported in the emergency medicine literature [9], and no reference to this association in emergency medicine textbooks [2,3] or electronic resources [404]. Although many occurrences of these cases should be evident to the endoscopist at the time of or shortly after the procedure, at least 8 documented cases have presented to the ED greater than 48 hours afterwards [10,11,21-23]. We have therefore elected to include these and other post-procedure cases in this review. Rupture of the spleen after other procedures appears to be very rare.

For the cases presented here with pathology in addition to the splenic rupture, there is a plausible causative relationship between the other pathology and the rupture for the vast majority. However, we have also included cases with a less clear patho-physiological relationship, such as the case reported 3 years after a pancreato-renal transplant [253], and that associated with viral hepatitis but no cirrhosis [113]. We acknowledge that the association in these cases may be coincidental and thus that these cases may better be classified as spontaneous. Although Wilson’s disease does not typically affect the spleen directly, the likely pathologic mechanism of the rupture in the case reported here is splenomegaly caused by portal hypertension [229].

We found only one case of delayed rupture of a normal spleen following trivial trauma reported in the literature in the last 60 years [392]. One other report of such a rupture in an enlarged but otherwise normal spleen [249], and reports of three others do not include information on the presence of splenic disease [393-395]. One additional case has been reported in a man 14 days after a mild fall, but the patient had also just been given heparin for a presumed myocardial infarction [396]. Given the dearth of publication in this area, the possibility remains that the associations observed in these reports are coincidental rather than causational. Regardless of the causative mechanism, these cases still meet the inclusion criteria for this review.

### Limitations

The primary goal of this paper is to highlight the occurrence of splenic rupture in patients without risk factors apparent on history. A secondary purpose is to document the diverse nature of illnesses that can present in this manner. However, we have not attempted to obtain papers that were not available to us either electronically, on paper at our library or through inter-library loan. We also have not attempted to have non-English or non-French language abstracts or papers translated. The possibility remains therefore that we have missed some rare causes of splenic rupture. In addition, while a general estimate of the relative frequency of different causes of splenic rupture can be made from the numbers reported here, the numbers for those that are frequently reported such as colonoscopy, malaria and lymphoma are likely underestimated because of publication bias. Conversely, the relative frequencies of rupture for rare or novel causes are likely over-estimated.

## Conclusions

Both traumatic and pathological rupture of the spleen are frequently reported in journals and documented in textbooks of emergency medicine. However, other causes of rupture are largely ignored in the emergency literature. We have documented a diverse range of patients for whom the presenting complaint for a disease was rupture of the spleen. We have also documented a number of medical procedures and medications that appear to have contributed to a rupture of the spleen, including some that have presented after the patients had been discharged from the facility conducting the procedure. Finally, we have documented several cases of trivial trauma associated with splenic rupture. Although these categories at first glance seem unrelated, they share the characteristic of having causes of rupture that would either be very subtle or completely unapparent on the presenting history, and are thus directly relevant to the practicing emergency physician. We hope that increased awareness of these phenomena will improve the ability of emergency clinicians to diagnose similar cases of splenic rupture in a timely fashion.

## Competing interests

The authors declare that they have no competing interests.

## Authors’ contributions

Both authors were involved in the literature search, review of the papers for inclusion, and the drafting of and revisions to the manuscript. KA takes full responsibility for the content. Both authors read and approved the final manuscript.

## Pre-publication history

The pre-publication history for this paper can be accessed here:

http://www.biomedcentral.com/1471-227X/12/11/prepub

## Author details

^1^Primary Healthcare Research Unit, Memorial University of Newfoundland, Health Sciences Centre, St. John’s, Newfoundland and Labrador, St Johns, Canada. ^2^Discipline of Emergency Medicine, Memorial University of Newfoundland, St. John’s, Newfoundland and Labrador, St Johns, Canada.^3^Discipline of Family Medicine, Memorial University of Newfoundland, St. John’s, Newfoundland and Labrador, St Johns, Canada. ^4^Department of Emergency Medicine, Dalhousie University, Halifax, NS, Canada.
